# RX-5902, a novel β-catenin modulator, potentiates the efficacy of immune checkpoint inhibitors in preclinical models of triple-negative breast Cancer

**DOI:** 10.1186/s12885-020-07500-1

**Published:** 2020-11-04

**Authors:** John J. Tentler, Julie Lang, Anna Capasso, Deog Joong Kim, Ely Benaim, Young B. Lee, Andrew Eisen, Stacey M. Bagby, Sarah J. Hartman, Betelehem W. Yacob, Brian Gittleman, Todd M. Pitts, Roberta Pelanda, S. Gail Eckhardt, Jennifer R. Diamond

**Affiliations:** 1 Division of Medical Oncology, School of Medicine, University of Colorado Anschutz Medical Campus, 12801 E 17th Ave, MS8117, Aurora, CO 80045 USA; 2 University of Colorado Cancer Center, Anschutz Medical Campus, 12801 E 17th Ave, MS8117, Aurora, CO 80045 USA; 3 Department of Immunology and Microbiology, University of Colorado Anschutz Medical Campus, Aurora, USA; 4 Dell Medical School, Department of Oncology, University of Texas at Austin, Austin, TX USA; 5 Rexahn Pharmaceuticals Inc., Rockville, MD USA

**Keywords:** RX-5902, Triple-negative breast cancer, p68, β-Catenin, PD-1 inhibitor, Immunotherapy, Humanized mouse models

## Abstract

**Background:**

Triple-negative breast cancer (TNBC) is an aggressive breast cancer subtype with limited systemic treatment options. RX-5902 is a novel anti-cancer agent that inhibits phosphorylated-p68 and thus attenuates nuclear β-catenin signaling. The purpose of this study was to evaluate the ability of β-catenin signaling blockade to enhance the efficacy of anti-CTLA-4 and anti-PD-1 immune checkpoint blockade in immunocompetent, preclinical models of TNBC.

**Methods:**

Treatment with RX-5902, anti-PD-1, anti-CTLA-4 or the combination was investigated in BALB/c mice injected with the 4 T1 TNBC cell line. Humanized BALB/c-Rag2^null^Il2rγ^null^SIRPα^NOD^ (hu-CB-BRGS) mice transplanted with a human immune system were implanted with MDA-MB-231 cells. Mice were randomized into treatment groups according to human hematopoietic chimerism and treated with RX-5902, anti-PD-1 or the combination. At sacrifice, bone marrow, lymph nodes, spleen and tumors were harvested for flow cytometry analysis of human immune cells.

**Results:**

The addition of RX-5902 to CTLA-4 or PD-1 inhibitors resulted in decreased tumor growth in the 4 T1 and human immune system and MDA-MB-231 xenograft models. Immunologic analyses demonstrated a significant increase in the number of activated T cells in tumor infiltrating lymphocytes (TILs) with RX-5902 treatment compared to vehicle (*p* < 0.05). In the RX-5902/nivolumab combination group, there was a significant increase in the percentage of CD4+ T cells in TILs and increased systemic granzyme B production (*p* < 0.01).

**Conclusions:**

Conclusions: RX-5902 enhanced the efficacy of nivolumab in a humanized, preclinical model of TNBC. Several changes in immunologic profiles were noted in mice treated with RX-5902 and the combination, including an increase in activated TILs and a decrease in human myeloid populations, that are often associated with immunosuppression in a tumor microenvironment. RX-5902 also was shown to potentiate the effects of checkpoint inhibitors of CTLA4 and the PD-1 inhibitor in the 4 T-1 murine TNBC model. These findings indicate that RX-5902 may have important immunomodulatory, as well as anti-tumor activity, in TNBC when combined with a checkpoint inhibitor.

## Background

Triple-negative breast cancer (TNBC) is an aggressive breast cancer subtype with limited systemic treatment options, in part due to a lack of expression of the estrogen receptor (ER), progesterone receptor (PR), and amplification of human epidermal growth factor receptor 2 (HER2) [1, 2]. TNBC makes up 10–20% of all breast cancers and is more common in younger women, African-American women, and those harboring BRCA1 mutations [3, 4]. TNBC can be further subdivided into molecular subtypes using gene expression profiling, however, the development of effective, targeted therapies for TNBC subtypes has been challenging [5, 6].

The emergence of immunotherapies targeting checkpoint proteins such as PD-1, PD-L1 and CTLA-4 has revolutionized the treatment of many cancers, including melanoma and non-small cell lung cancer, where these therapies lead to a meaningful improvement in the overall survival of patients with metastatic disease [7–10]. In metastatic TNBC, the addition of the PD-L1 inhibitor atezolizumab to chemotherapy with nab-paclitaxel improved progression-free survival in the intent-to-treat population and the PD-L1-positive subset, leading to the FDA-approval of this combination in patients with PD-L1-positive metastatic TNBC [11]. Despite these promising results, the median progression-free survival with combination therapy in the PD-L1-positive subgroup was only 7.5 months, highlighting the critical need to enhance the response to immune checkpoint inhibitors in TNBC and increase the proportion of responding patients and the duration of benefit.

Inhibitors of the Wnt/β-catenin signaling pathway have the potential to improve the response to immune checkpoint inhibitors given the role of Wnt signaling in proliferation, maturation and differentiation of dendritic and T cells [12, 13]. High levels of nuclear β-catenin are associated with low tumor-infiltrating lymphocytes (TILs) and poor response to immune checkpoint inhibitors [14]. In breast cancer, mutations in Wnt pathway components are uncommon, however, activation of Wnt signaling is observed in over half of breast cancers and associated with inferior overall survival [15].

The p68 RNA helicase is a prototypical member of the DEAD-box family of nuclear RNA helicases with roles in pre-mRNA, rRNA and miRNA processing and ribosome biogenesis [16]. p68 can be phosphorylated at Y593 by growth factors, including platelet-derived growth factor (PDGF) acting through c-Abl signaling, promoting oncogenesis through enhanced β-catenin nuclear localization [17]. p-p68 functions as a nuclear chaperone for β-catenin, promoting nuclear translocation and activation of cyclin D1, c-JUN and c-MYC [17, 18]. In this role, p-p68 regulates cellular proliferation, malignant transformation, epithelial to mesenchymal transition (EMT) and cell migration pointing to a vital role in oncogenesis and cancer progression [17, 19]. RX-5902 is a first in-class, orally bioavailable, small molecule inhibitor of phosphorylated-p68 (p-p68) that decreases nuclear localization of β-catenin in preclinical models of solid tumors [20–22]. RX-5902 interacts with Y593 p-p68 and inhibits β-catenin-dependent ATPase activity and has minimal impact on the RNA-dependent ATPase activity of p68 [20]. RX-5902 exposure results in potent antiproliferative activity in TNBC cell lines and tumor growth inhibition in cell line xenograft and patient-derived tumor xenograft models [22]. Additionally, RX-5902 treatment in preclinical TNBC models results in a decrease in nuclear β-catenin and cellular levels of MCL-1 with induction of apoptosis, confirming target engagement [22].

The purpose of this study was to evaluate the combination of RX-5902 with CTLA-4 and PD-1 immune checkpoint inhibitors in immunocompetent mouse models of TNBC to determine if inhibition of p-p68 may potentiate the activity of immunotherapy. Correlative studies were performed in a human immune system mouse model to evaluate effects on human TILs and immune activation markers.

## Methods

### Drugs

RX-5902 was provided by Rexahn, Inc. (Rockville, MD) [22]. The drug was prepared in sterile miliQ H_2_O for in vivo dosing. The anti-mouse CTLA-4 (CD152, clone 9D9) and anti-mouse PD-1 (CD279, clone RMP1–14) monoclonal antibodies were obtained from Bio X cell (West Lebanon, NH). Nivolumab (anti-human PD-1) was obtained from the University of Colorado Hospital Pharmacy.

### Cancer cell lines

The human MDA-MB-231 TNBC cell line was purchased from American Type Culture Collection and cultured in DMEM supplemented with 10% FBS, 1% penicillin–streptomycin, 1% MEM nonessential amino acids and 1% normocin. Cells were harvested during exponential growth and resuspended in a 1:1 mixture of DMEM supplemented with 10% FBS, 1% PenStrep and 1% non-essential amino acids and Matrigel (BD Biosciences) prior to injection into mice. Cells were authenticated by the Barbara Davis Center for Childhood Diabetes Core and tested for the absence of mycoplasma every 3 months. The murine 4 T1 TNBC cell line was obtained from Crown Bioscience Inc. (Taicang, China) and cultured in RPMI media supplemented with 10% FBS. All cells were grown at 37 °C with 5% CO_2_ in an incubator.

### Animal studies

All animal studies were performed in accordance with the NIH guidelines for the care and use of laboratory animals in a facility accredited by the American Association for Accreditation of Laboratory Animal Care with approval by the Institutional Animal Care and Use Committee prior to initiation of experiments. Tumor cell inoculations were performed under isoflurane anaesthesia via regulated administation machines. Animals were euthanized via CO2 and cervical dislocation. Approximately 60 animals were utilized in these studies.

### 4 T1 murine TNBC xenograft model

Five- to six-week-old BALB/c mice were housed in groups of five per cage and allowed 1 week of acclimation prior to handling. 4 T1 cells were harvested during logarithmic growth and 3 × 10^5^ cells in 100 μl of PBS were injected subcutaneously in the right lower flank. When tumors reached a median tumor volume of ~ 90 mm^3^, mice were randomized into treatment arms: vehicle, RX-5902 60 mg/kg by oral gavage daily, anti-PD-1100 μg/ mouse i.p. twice a week × 4 weeks or the combination. Tumor growth inhibition (TGI) was calculated using the following formula: TGI (%) =100 x (1- *V*_t_ / *V*_vc_)) where *V*_t_ and *V*_vc_) is the mean tumor volume of the treated and vehicle control groups, respectively. The experiment was repeated as above and mice were randomized to following treatment arms: vehicle, RX-5902 35 mg/kg by oral gavage daily, RX-5902 35 mg/kg plus anti-CTLA-4 (5 mg/kg) i.p. twice a week × 3 weeks, RX-5902 (75 mg/kg), or RX-5902 (75 mg/kg) plus anti-CTLA-4 (5 mg/kg) i.p. twice a week × 3 weeks. The 4 T1 experiments were performed by Crown Bioscience Inc. (Taicang, China).

### Stem cell isolation for the generation of the humanized mice

CD34+ hematopoietic stem cells (HSCs) were isolated using magnetic CD34+ Miltenyi beads from PBMCs prepared from clinically-rejected cord blood units from the University of Colorado Cord Blood bank at Clinimmune Labs (Aurora, CO) and expanded in short-term cultures with IL6, SCF and Flt3L as previously described [23]. CD34+ cells were frozen in 90% FCS/10% DMSO and stored at − 80 °C.

### Human immune system (hu-CB-BRGS) mice and hematopoietic chimerism evaluation

To generate human immune system (HIS) mice, 0.2–0.6 × 10^6^ expanded and thawed CD34+ cells were injected into neonate BRGS (BALB/c *Rag2*^nul*l*^*IL2Rγ*^null^*Sirpa*^NOD^) pups that had been irradiated with 300 rad 2–6 h prior to injection [18]. Mice were injected in the facial vein or liver, as previously described [18]. Mice were weaned at 3–4 weeks of age and analyzed for human chimerism at 10 and 16 weeks of age by staining of PBMCs, isolated from retro-orbital bleeds, to determine human (hCD45+), T (hCD3, hCD8, hCD5) and B (hCD20) cell chimerism. Human chimerism was defined as % hCD45+/(% hCD45+ + % mCD45+), the chimerism of the total (human+mouse) hematopoietic system. The mice were sorted into experimental groups based on overall human and T cell chimerism detected in the blood at the later time point.

### Hu-CB-BRGS mice treatment studies

At 19 weeks post engraftment, 5 × 10^6^ MDA-MB-231 cells were injected into the left and right flanks of hu-CB-BRGS mice. When the average tumor size reached a volume of ~ 150–300 mm^3^, mice were randomized into treatment groups: vehicle control, RX-5902 15 mg/kg by oral gavage daily, nivolumab (anti-human PD-1) 15 mg/kg i.p. twice weekly, or the combination according to levels of human and T cell chimerism as previously described [18]. Tumor size was evaluated twice weekly by caliper measurements and the tumor volume was calculated (tumor volume = (length × width^2^) × 0.52). Tumor growth inhibition (TGI) was calculated using the following formula: TGI (%) =100 x (1- *V*_t_ / *V*_vc_)) where *V*_t_ and *V*_vc_ is the mean tumor volume of the treated and vehicle control groups, respectively. Treatment was continued until tumors reached a single volume of 2000 mm^3^ or a total volume of 3000 mm^3^/mouse. Exponential growth rate was calculated using the formula: Y=Y0*exp.(k*X) where Y0 is the Y value when X (time) is zero and k is the rate constant expressed in reciprocal of the X axis time units (days).

### Immune correlatives in the hu-CB-BRGS mice experiments

At sacrifice, bone marrow, lymph nodes (LN), spleen and tumors were harvested for flow cytometry analysis of human immune cell characteristics. Single cell suspensions were prepared using mechanical disruption with frosted glass slides, flushing of tibias and femurs and mechanical disruption with razor blades followed by Liberase DL digestion for LN and spleen, bone marrow and tumors, respectively as previously described [18] . Filtered single-cell suspensions were stained as previously described [18] Flow cytometry was performed with constant rates and times on a Cyan analyzer (Dako Cytomation) at the University of Colorado Cancer Center flow cytometry core facility. FlowJo software was used for data analysis.

At sacrifice, a portion of the dissected tumor was fixed in formalin, paraffin-embedded and prepared for multispectral imaging on Vectra 3.0 Automated Quantitative Pathology Imaging System (Perkin Elmer) as previously described [18]. In brief, four micron sections of formalin-fixed tumor tissues were mounted onto glass slides and sequentially stained for human CD3 and pan-cytokeratin on a Bond RX autostainer (Leica) and HRP-reactive OPAL fluorescent technology (Opal 540, Perkin Elmer). Whole slide scans were collected using the 10x objective and approximately 10 regions were selected for multispectral imaging with the 20x objective. The multispectral images were analyzed with inForm software (Perkin Elmer).

### Statistical analysis

Treatment groups were compared by ANOVA parametric analysis of the means using a commercially available statistical program (Prism GraphPad 8.3.0). The comparisons between sensitivity of treatment and vehicle groups in vivo were analyzed using an unpaired two-tailed parametric *t-*test with Welch’s corrections when appropriate (Prism Graph Pad 8.3.0).

## Results

### RX-5902 enhances the anti-tumor activity of anti-PD-1 and anti-CTLA-4 antibodies in the 4 T1 immunocompetent murine model of TNBC

**Fig. 1:**
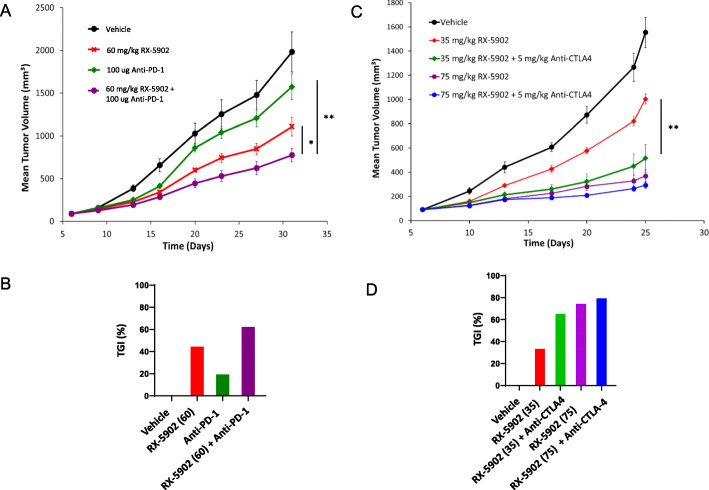
**a**-**b** Treatment of 4 T1 TNBC xenograft model with vehicle control, RX-5902 (60 mg/kg), anti-mouse PD-1 (100 μg/mouse) or the combination. TGI compared to vehicle: RX-5902 60 (TGI = 44%; *p* = 0.007), anti-mouse-PD-1 (TGI = 21%; *p* = 0.173), combination (TGI = 61%; *p* = 0.001). Combination compared to RX-5902 alone and anti-mouse-PD-1 alone (*p* = 0.032 and *p* = 0.001, respectively). **c**-**d** Treatment of 4 T1 TNBC xenograft model with vehicle control, RX-5902 (35 mg/kg), RX-5902 (75 mg/kg), RX-5902 (35 mg/kg) plus anti-CTLA-4 (5 mg/kg) or RX-5902 (75 mg/kg) plus anti-CTLA-4 (5 mg/kg) resulted in TGI at day 25 compared to vehicle control: TGI 35%, *p* = 0.006; TGI 76%, *p* < 0.001; TGI 67%, *p* < 0.001, TGI 76%, *p* < 0.001. Combination of RX-5902 (35 mg/kg) plus anti-CTLA-4 (5 mg/kg) compared to RX-5902 alone (*p* = 0.007). * *p* < 0.05, **p < 0.01

Given the ability of β-catenin to modulate the anti-cancer immune response, we sought to determine if treatment with RX-5902 could enhance the anti-tumor activity of anti-mouse-PD-1 or anti-mouse-CTLA-4 monoclonal antibodies in an immune-competent BALB/c, 4 T1 mouse model of TNBC [14]. As depicted in Fig. 1a-b, single agent RX-5902 60 mg/kg treatment resulted in significant antitumor activity compared to vehicle control (TGI = 44%; *p* = 0.007), whereas single agent anti-mouse-PD-1 was minimally active (TGI = 21%; *p* = 0.173). However, the combination treatment of RX-5902 with anti-mouse-PD-1 resulted in significant antitumor activity (TGI = 61%; *p* = 0.001) compared to vehicle control and to the RX-5902 and anti-mouse-PD-1 alone treatment groups (*p* = 0.032 and *p* = 0.001, respectively).

As shown in Fig. 1c-d, single agent treatment with RX-5902 35 mg/kg and 75 mg/kg treatment resulted in significant, dose proportional antitumor activity compared to vehicle control (TGI = 35%; *p* = 0.006 and TGI = 76%; *p* = 0.001, respectively). The combinations of RX-5902 (35 mg/kg and 75 mg/kg) and anti-mouse-CTLA-4 were also efficacious compared to vehicle control (TGI = 67%; *p* = < 0.001, TGI = 81%; *p* = < 0.001, respectively). The combination of lower dose RX-5902 (35 mg/kg) plus anti-CTLA-4 resulted in greater tumor growth inhibition compared to RX-5902 (35 mg/kg) alone (*p* = 0.007). All treatments were well-tolerated and no severe weight loss or abnormal clinical signs were observed in the mice these studies.

### RX-5902 potentiates the activity of anti-PD-1 in a humanized mouse model of TNBC

**Fig. 2:**
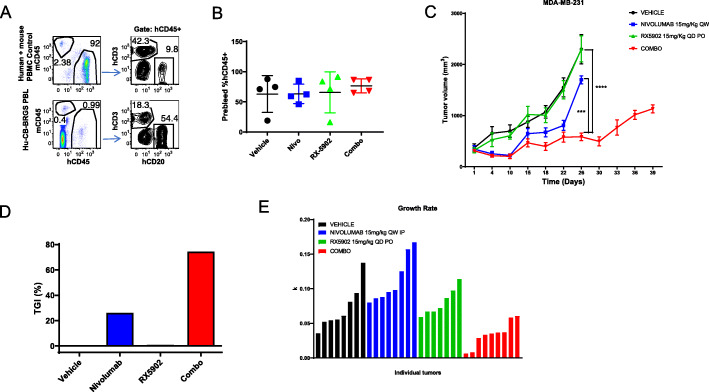
**a** Flow cytometry gating strategy for detecting presence of human immune cells in blood of hu-CB-BRGS mice 16 weeks post engraftment and prior to tumor cell inoculation. **b** Percentage of human CD45+ cells in blood of hu-CB-BRGS mice plotted according to their distribution into treatment groups. **c** Tumor growth curves of MDA-MB-231 TNBC cell line xenograft tumors in hu-CB-BRGS mice at the indicated doses. Vehicle, Nivo, and RX-5902 arms were ended at Day 26 due to tumor size limits. Treatment was continued for the Combo arm due to their increased survival compared to the other groups. **d** Percent tumor growth inhibition (%TGI) for the indicated treatment groups. **e** Tumor growth rates for individual tumors in the indicated treatment groups over the duration of the treatment time. ****p < 0.0001; ***p < 0.001; *p < 0.05

We used the humanized (hu-CB-BRGS) mouse MBA-MD-231 xenograft model to assess the efficacy of combining RX-5902 with the anti-human-PD-1 antibody nivolumab. This system allowed evaluation of both the anti-tumor activity and the impact on the human anti-tumor immune response of RX-5902, nivolumab and the combination. Mice were randomized to treatment groups based on their human hematopoietic chimerism and the percentage of human hematopoietic (CD45+), and human T (CD3+) and B (CD20+) subsets in blood were also well-represented across the treatment groups prior to initiation of treatment (Fig. 2a, b). Submaximal doses of nivolumab and RX-5902 as single agents resulted in modest inhibition of tumor growth, however, the combination resulted in a statistically significant inhibition and reduction in tumor doubling time (Fig. 2c) Tumor growth inhibition (TGI) was calculated at 26% for Nivo alone; − 0.28% for RX-5902 alone; and 74.5% for the combo, compared to vehicle (Fig. 2d). Additionally, tumor growth rate, as measured by volume doubling time, was also greatly reduced in the combo treatment arm (Fig. 2e). Treatment in the combination group was continued for approximately 2 weeks longer than the other treatment arms to observe long-term efficacy (Fig. 2c). The observed increase in tumor growth during this period may indicate an escape of the tumor from immune control, potentially due to loss of human chimerism.

### Immune cell characterization in the hu-CB-BRGS MDA-MB-231 xenograft model treated with RX-5902, nivolumab or the combination

We performed a comprehensive analysis of human immune cell characteristics using flow cytometry of single cell suspensions isolated from the spleen, LNs, bone marrow and tumor tissue obtained at the time of sacrifice from all treatment arms (Fig. 2c). The combination treatment group was sacrificed approximately 2 weeks after the other treatment groups based on the parameters for sacrifice based on tumor size and animal weights.

**Fig. 3:**
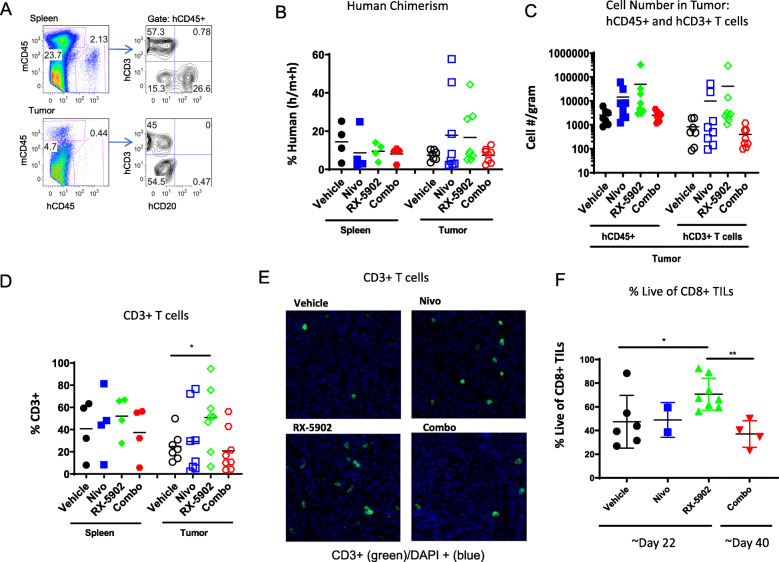
Human immune subsets in spleens and tumors of hu-CB-BRGS MDA-MB-231 bearing mice at the end of study. **a** Flow cytometry panel for identification of mouse (mCD45) and human (mCD45) hematopoietic cells, and T (CD3) and B (CD20) cells among the hCD45+ population (right), in the spleen (top) and tumor (bottom). **b** Percentage of human (hCD45+ of (mCD45^+^ + hCD45^+^)) cells in the spleens and tumors. **c** T cell (CD3+) numbers and frequencies and **d** among the hCD45+ cells. **e** Representative fields from tumors collected at end of study from the indicated treatment arms stained with anti-CD3 antibodies (green) and DAPI and imaged using the Vectra 3.0 system. **f** Frequency of live cells among CD8+ human (hCD45+) cells in tumors. Each dot represents data from an individual spleen, bone marrow or tumor from mice receiving either no drug (Vehicle), nivolumab alone (Nivo), RX-5902 alone, or combination of nivolumab and RX-5902 (Combo) treatment. Lines represent arithmetic means. *p*-value: * < 0.05

Human hematopoietic chimerism was evaluated at the end of study in spleen and tumor tissue. Figures 3a, b depict the % human (h) hematopoietic cells in these tissues. As expected, there was no significant difference in the frequency of hCD45+ cells in the spleen across treatment groups (Fig. 3b). We did observe a few tumors in the single agent arms with increased frequencies of hCD45+ cells, however, there was no statistically significant difference across the treatment groups (Fig. 3b).

We analyzed the overall composition of the human immune system by cell type in the spleen and tumor tissue to determine if the effects observed were systemic (i.e. present in spleen and tumor tissue) or present only in the tumor microenvironment. Among the hCD45+ cells, we determined the fraction of T cells (CD3) and observed a statistically significant increase in absolute number and frequency of CD3+ T cells in the tumors of the RX-5902 treated mice compared to vehicle (Fig. 3c-d). An increase in CD3+ T cells in the tumor microenvironment was also observed using immunofluorescence (IF) performed on fixed tumor tissues (Fig. 3e). We further characterized the TILs and observed a statistically significant increase in the frequency of live CD8+ TILs in the RX-5902 treated tumors suggesting a functional advantage for these cells treated with RX-5902 (Fig. 3f). The increase in CD3+ T cells in the tumor tissue that was observed consistently with RX-5902 treatment was not seen in the group treated with nivolumab and RX-5902. There are various potential explanations for these findings, including differences in timing of animal sacrifice, immune cell exhaustion, and loss of chimerism over time.

**Fig. 4:**
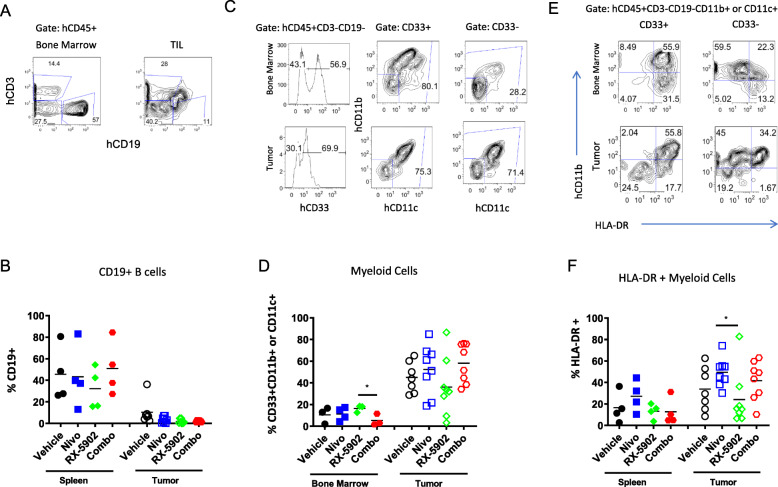
**a** Flow cytometric gating strategy depicting identification of T (CD3) and B (CD19+) cells among hCD45+ hematopoietic cells in the spleen (left) and tumor (right). **b** Frequencies of B (CD19+) B cells in each mouse in spleen and tumors. **c** Gating strategy to detect myeloid cells, defined as CD33+, CD11b + and/or CD11c+, among the hCD45+ cells in bone marrow and tumors. **d** Frequencies of myeloid cells in each hu-CB-BRGS mouse in bone marrow and tumor. **e** Representative flow cytometry analysis depicting expression of HLA-DR MHC Class II molecules on hCD45+ myeloid cells in spleen (top) or tumor (bottom). **f** Frequencies of HLA-DR+ among myeloid cells in the spleen and tumor for each hu-CB-BRGS mouse. Each dot represents data from an individual spleen, bone marrow or tumor from mice receiving either no drug (Vehicle), nivolumab alone (Nivo), RX-5902 alone, or combination of nivolumab and RX-5902 (Combo) treatment. Lines represent arithmetic means. *p*-value: * < 0.05

The frequency of B cells was low regardless of treatment arm in tumor tissue as compared to the spleen. (Fig. 4a-b). The frequency of human myeloid cells defined by the expression of either CD33, CD11b or CD11c in CD3, CD19-negative, hCD45+ cells was higher in tumor tissue as compared to bone marrow (Fig. 4c-d). We observed a decrease in the frequency of myeloid cells in the tumors treated with RX-5902, however, this did not reach statistical significance (Fig. 4d). Myeloid cells in tumors are often associated with an immunosuppressive tumor microenvironment and low expression of HLA-DR. We used the expression of the HLA-DR class II molecule as a relative readout for the ability of the human myeloid cells to present antigen to the T cells, and thus function in a more immunogenic manner (Fig. 4e-f). We found that human myeloid cells in the tumors from nivolumab-treated mice had higher frequencies of HLA-DR+ cells. There was a statistically significant increase in the nivolumab-treated mice compared to vehicle (*p* < 0.05) and a trend with combination treatment. Although this marker alone does not definitively identify these cells as non-myeloid-derived suppressor cells (MDSCs), the higher expression of the class II molecule suggests these myeloid cells are in a more immunogenic state.

### Human immune cell functional analysis

**Fig. 5:**
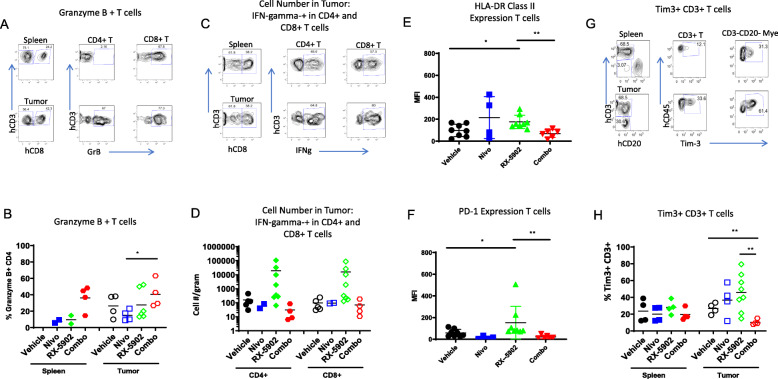
Human T cell function in spleens and tumors of hu-CB-BRGS MDA-MB-231 bearing mice. Flow cytometry gating strategy for measurement of **a** Granzyme B, and **c** IFNγ producing CD4 and CD8 T cells in the spleen or tumors and the calculated frequencies of **b** Granzyme B, and **d** relative cell numbers, per g of tumor, of IFNγ producing CD4 and CD8 T cells in spleens and tumors of each hu-CB-BRGS mouse. **a** represents sample from Combo treated mice, and **c** from RX-5902 treated mice. Expression (as determine by mean fluorescence intensity, MFI) of **e** HLA-DR activation, and **f** inhibitory PD-1 molecules on human T cells in tumors. **g** Flow cytometry analysis measuring frequency of Tim3+ cells among T cells in the spleens and tumors. **h** Frequency of Tim-3+ cells among human T cells in spleens and tumors of hu-CB-BRGS tumor-bearing mice. For all graphs, each dot represents data from an individual spleen or tumor from mice receiving either no drug (Vehicle), nivolumab alone (Nivo), Rexahn-5902 alone (RX-5902) or a combination of nivolumab and Rexahn-5902 (Combo) treatment. Lines represent arithmetic means. *p*-values: * < 0.05, ** < 0.01

In addition to characterizing the make-up of immune cells present in tumor and peripheral tissues, we investigated functional markers present on immune cells associated with either anti- or pro-tumor immune responses. Granzyme B (GrB) is a cytotoxic molecule secreted by activated T cells and natural killer (NK) cells [24, 25]. We observed an increase in the frequency of GrB+ CD4 and CD8 among T cells in the tumors from mice treated with the combination of RX-5902 and nivolumab compared to either agent alone (Fig. 5a-b). Although the frequency of T cells was lower in the tumors treated with RX-5902 plus nivolumab (Fig. 3c-d), the anti-tumor activity of this combination was greater (Fig. 2c) and there was a higher frequency of GrB+ activated T cells in tumors from the combination group.

In addition to GrB, the secretion of the cytotoxicity-associated IFNγ cytokine by CD8 T cells is widely reported to be associated with a favorable response to immune checkpoint blockade [26]. Analysis of IFNγ secretion by either CD4 or CD8 T cells showed increased production with only RX-5902 treatment alone in tumor tissue (Figs. 5c-d). MHC Class II is upregulated upon activation of T cells. Consistent with increased functionality of T cells in tumors from RX-5902 treated mice, we observed an increase in HLA-DR MHC Class II expression among the T cells in the tumor from RX-5902 treated mice (Fig. 5e). The low frequency in the combination treated mice could be explained either by an inhibition of this mechanism with nivolumab, which is in opposition to other reports, or a kinetic effect of the study.

Finally, we measured the expression of inhibitory molecules PD-1 and Tim-3 as a further indicator of immune status. Both PD-1 and TIM-3 are inhibitory receptors on activated and/or exhausted T cells. Specifically, TIM-3 is an immune inhibitory receptor associated with highly differentiated, IFNg+ T cells and exhausted T cells [27, 28]. RX-5902 treatment as a single agent resulted in the highest expression of both PD-1 and Tim-3 on T cells from all treatment groups (Figs. 5f-h). This increased PD-1 and Tim-3 is indicative of more activated and differentiated T cells, and could represent an increase in targetable T cells populations for checkpoint inhibition with RX-5902 treatment. Of note, we were not able to determine PD-1 expression on T cells of mice treated with nivolumab as this monoclonal antibody targets and blocks PD-1.

### Impact of treatment on tumor immunogenicity

**Fig. 6:**
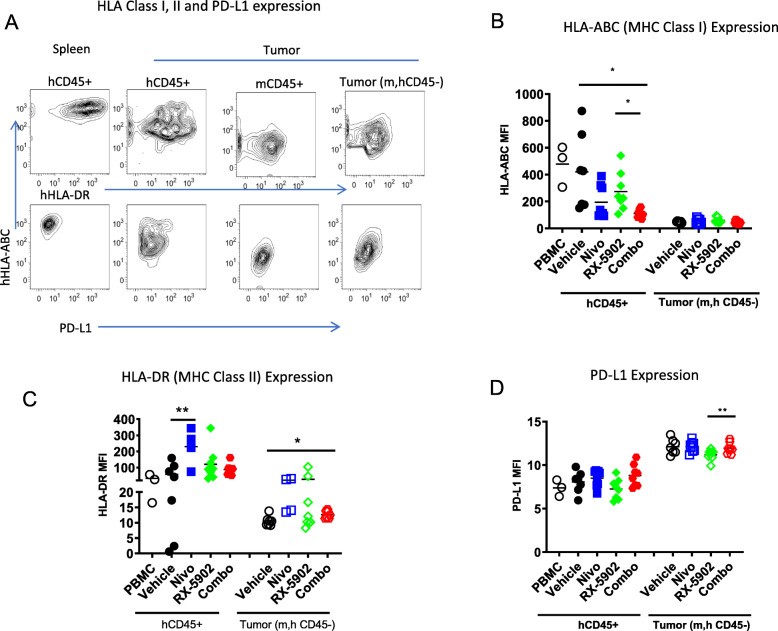
**a** Expression of human MHC Class I (HLA-ABC), Class II (HLA-DR) and PD-L1 on cells in MDA-MB-231 tumors from hu-CB-BRGS mice. Tumors extracted from hu-CB-BRGS mice were prepared into single cell suspensions and stained for mCD45, hCD45 and Epcam. The mean fluorescence intensities (MFIs) of HLA-ABC MHC Class I **b**, HLA-DR MHC Class II **c** and PD-L1 **d** were determined on both the human hematopoetic (hCD45+) and the tumor cells (m,hCD45-). Expression of these molecules on PBMCs were included as a positive control for each graph. Each dot represents data from an individual tumor from mice receiving either no drug (Vehicle), nivolumab alone (Nivo), Rexahn-5902 alone (RX-5902) or a combination of Nivolumab and RX-5902 (Combo) treatment. Lines represent arithmetic means. *p*-values: * < 0.05, ** < 0.01

A common mechanism for tumors to escape immune surveillance is to downregulate HLA molecules and upregulate immunosuppressive molecules such as PD-L1 [29]. Thus we again used flow cytometry to analyze expression of MHC Class I (HLA-ABC) and Class II (HLA-DR),as well as PD-L1, on non-hematopoietic tumor cells (mCD45-, hCD45-). We used the expression of these molecules on hCD45+ cells in the spleens and human PBMCs as controls (Fig. 6a-d). As expected, we found very low levels of both Class I and Class II HLA molecules on tumor cells in all mice, with a modest, yet significant, upregulation of HLA-DR on tumor cells in combination-treated mice relative to controls (Fig. 6b-c). However, PD-L1 expression was higher on the tumor cells relative to control human PBMCs or hCD45+ cells in the spleen or tumors of the hu-CB-BRGS tumor-bearing mice (Fig. 6d). Notably, the expression of the immunosuppressive PD-L1 molecule was lower on tumor cells in RX-5092 treated mice (Fig. 6d).

## Discussion

In this study, we explored inhibition of the Wnt/β-catenin signaling pathway as a strategy to potentiate the anti-tumor activity of immune checkpoint inhibition in immunocompetent models of TNBC. We utilized the novel inhibitor of p-p68, RX-5902, due to its ability to block the coactivator function of p-p68 and prevent p-p68-mediated nuclear shuttling of β-catenin, thereby negating its transcriptional activities [20, 22]. The Wnt/β-catenin pathway is a well-characterized driver of several cancers, a promoter of cancer progression and mediator of resistance to checkpoint inhibitors through its dysregulation of tumor-immune cycle [14]. Specifically, β-catenin signaling directly affects regulators critical for T-cell tumor infiltration, and antitumor activities of effector T cells, T helper cells and regulatory T cells. Given the association of elevated β-catenin signaling with low TIL infiltration and in resistance to checkpoint inhibitors, we sought to determine if RX-5902, when combined with anti-CTLA4 and PD-1 monoclonal antibodies, could enhance anti-tumor and immunologic responses in a preclinical models of TNBC. Towards this end, we utilized the 4 T1 mouse model of TNBC as well as a hu-CB-BRGS humanized mouse model bearing human MDA-MB-231 TNBC xenograft tumors which would allow for end of treatment immune analyses to further elucidate the mechanisms of RX-5902 action.

Our intention in the humanized mouse studies was to evaluate the human immune populations in the tumor in an attempt to correlate changes with treatments and/or immune responses (tumor growth). Our hu-CB-BRGS HIS mouse model, similar to many humanized mouse models, generates a multi-lineage human immune system, with caveats [30]. The lymphocyte lineage is overrepresented, initially by B cells and followed by T cells. Thus these lineages are easy to detect and interrogate. Human myeloid cells do develop, but more variably and less robustly, and are most abundant in the bone marrow and non-lymph organs, such as liver and lung, physiologically appropriate locations. NK cells that develop are immature and non-functional, in the absence of exogenous IL-15, as reported in several publications regarding HIS mice [31]. Given these limitations, and the abundant role of T cells following checkpoint blockade immunotherapy treatments in both mouse and human cancers, we focused our analysis on the lymphocytes, and more specifically functional assays for the T cells. We enumerated the frequency of both human B and myeloid cells as well, however we did not interrogate their subsets and functions. We also did not evaluate the NK cell populations, given that this is a minor, non-functional population of the immune system in this model. Since immature NK cells do not express CD11b [32], the NK population in our mice would be represented in the Lin- (CD3-CD19-CD33-CD11b-CD11c-) fraction, which is very minor in our system. The human myeloid population consists of numerous subtypes, including monocytes, macrophages and multiple types of dendritic cells. As with all immune cell subsets, the function of each can be immune-activating or suppressing, dependent on environmental context. In tumors, the myeloid lineage is most often associated with immunosuppression with a large population of tumor associated macrophages (TAMs, M2) and MDSCs. The flow cytometric phenotypes of these lineages are still disputed in the literature; however, a common, albeit not exclusive, feature of immunosuppressive myeloid cells is a lower expression of HLA-DR. Therefore, we calculated the HLA-DR high frequency as an indication of ability to present antigen. Notably, we did not detect significant alterations in the frequency of myeloid lineage cells in any treatment group.

In dosing studies, we found that in both models, RX-5902 demonstrated anti-tumor activity on its own but this was significantly enhanced when combined with checkpoint inhibitors. The tumors in the hu-CB-BRGS MDA-MB-231 bearing mice were well-infiltrated with human immune cells, unlike other “cold” tumors we and others have observed with the use of this model [18, 33]. The major infiltrates are T cells and myeloid cells, with very few B cells consistent with other reports [18, 34].

Treatment with RX-5902, both alone and in combination with nivolumab, resulted in changes in human immune components in MDA-MB-231 tumors that are largely consistent with reports in other human and mouse studies involving inhibition of β-catenin signaling. Specifically, we observed increases in total T cells and CD8+ T cell infiltration with RX-5902. In our study these RX-5902 exposed T cells, both CD4 and CD8, produced increased IFNγ, as observed in other reports with β-catenin inhibitors [14, 35]. However, this T cell increase did not correlate with tumor growth, suggesting this response was suboptimal as a single agent. Notably, in our system we also observed consistent increases of Granzyme B production, again by both CD4 and CD8 T cells, when the RX-5902 is combined with nivolumab. Further investigation is required to understand if the Granzyme B pathway is related to the decreased tumor growth, however the cytotoxic nature of this pathway is intriguing. Indeed we have seen this correlation in other humanized mouse immunotherapy studies [36] and there are reports that Granzyme B predicts immunotherapy response in other models [30]. We also quantified the Treg population by measuring co-expression of CD25 and FoxP3, but in contrast to other reports [14, 35], we did not detect changes in the frequencies among treatment groups of this clearly detectable population that represented on average 3.5% of human TILs (Supplementary Fig 1). A previous report studying PRI-724, a CBP/β-catenin inhibitor, in a mouse model of colorectal cancer, observed increases in the Treg population with drug treatment, suggesting that a decrease in Tregs is not a consistent finding with β-catenin inhibition [37]. Our analysis of the myeloid populations showed that although they are underrepresented in general in humanized mice, we did observe substantial representation of these cells in tumors. An increase in CD103+ DC in tumors has been observed in some tumors with β-catenin inhibition. While we did not look at this population specifically, we did observe an increased trend in MHC Class II (HLA-DR) expression on tumor myeloid cells with nivolumab or combination therapy. The higher Class II expression is often associated with increased antigen presentation and inflammatory myeloid cells, consistent with increased antigen presentation by DCs. One interesting result from our study is an increased expression of inhibitory receptor TIM-3 on the myeloid cells in the tumor with RX-5902 alone, indicating that this co-receptor is a possible combination target. In contrast to the changes we observed in treated immune cells, we did not observe changes in the expression of MHC Class I or II molecules or PD-L1 inhibitory receptor on the tumor themselves, suggesting that in our model the major influence of the β-catenin inhibitor is on the immune system and less so on the tumor. We of course cannot rule out that other changes, that were not measured, occurred as well. Although we observed T cell phenotype, activation and functional differences among treatment groups, and not changes in either B cell or myeloid populations, we cannot rule out changes among myeloid subsets with treatments, as we did not examine these populations in depth.

Human immune system mice do not fully recapitulate the immune environment of human patients. The immune system takes time to develop and T cells often do not appear until months after cord blood engraftment. There is also variability among chimerism. To take these issues into account, we verified the presence of T cells in blood of mice before tumor injection and randomized our treatment groups according to overall human and T cell chimerism. In addition, we ensured the development of LNs at end of study and splenic chimerism as inclusion criteria. Furthermore, the kinetics of these humanized mice experiments are important and may affect the results. In our studies, we harvested mice over a three-week time period, for both practical reasons and to acquire as much tumor growth data as possible. Because the MDA-MB-231 tumors in the combination arm grew at a far slower rate, they were allowed to continue in the study for a longer time period before harvest and analysis, which may explain some discrepant data observed, such as lower tumor T cell numbers. Since the tumor-immune interface is a dynamic process, fluctuating between activated and suppressed responses, it is possible that our snap-shot of the immune system, as for all similar immune interrogations following treatments in mice or humans, is affected by these different time points. It is well-appreciated that T cell activation is a dynamic process, beginning with upregulation of activation markers, rapid proliferation of antigen-specific T cells, followed by exhaustion of T cells that can include activation-induced cell death. In this experiment, there were fewer T cells in the combination-treated animals, with a higher death rate as seen by lower frequency of live cells among the CD8+ T cells. The increased number of T cells, frequency of live CD8+ T cells, and expression of activation markers HLA-DR and Tim-3 expression in the RX-5092 cohort suggest more activated T cells in this group. However, this could also indicate a T cell response earlier in the process. Given the dynamic changes in the constitution of immune cells in the tumor microenvironment over time, this difference in timing of obtaining tumor tissue for the planned immune correlatives hinders clear interpretations as to whether the T cells, or other immune subsets, are responsible for the tumor response. In future studies efforts will be made to minimize these differences.

The final outcome of an immune response such as tumor growth inhibition is a highly coordinated process, involving integration of both positive and negative signals across multiple, interacting cell receptors and cell types, including the antigen presenting cell and the responding lymphocyte. Even very sophisticated analyses such as mass cytometry, RNA-seq and ATAC-seq data of the immune populations within tumors (or blood) are at best correlative to the tumor response, at the moment in time in which the immune cells are being evaluated. Attempts to understand mechanism of tumor rejection by an immune response is always muddled with these limitations. However, the evaluation of the immune phenotypes, characteristics and functions within a model, and identifying differences among treatment cohorts, can direct further studies. Our studies suggest that RX-5092 may alter T cell responses, although we cannot say whether this is a direct effect on the T cell or through antigen presenting cells.

## Conclusions

Together, these preclinical models allowed us to demonstrate that RX-5902, in addition to its direct anti-tumor activity, can induce positive alterations in the tumor immunologic environment that may allow for enhanced responses to immunotherapies. This may be due to its effects on decreased β-catenin signaling, though additional effects on other p68 targets is also possible. These preclinical results support a planned Phase 2 clinical trial combining RX-5902 with nivolumab in patients with TNBC, which will include biological correlates designed to identify biomarkers of response that may inform improved patient selection strategies for this population.

## Supplementary information

**Additional file 1: Figure S1.** No difference observed in frequency of T regulatory cells (CD25 + FoxP3+) among human CD4+ T cells in the tumors of control or treated mice. A) Representative flow plots illustrating detection of Tregs by CD25 and FoxP3 and B) frequency of Tregs among CD4+ T cells in the lymph nodes (LN) and tumors (TIL) for each mouse, according to treatment. Bars represent arithmetic means.

**Additional file 2: Table S1.** Antibodies used in these studies. Antibody Sources, Clone ID and Fluorochromes are as indicated.

## Data Availability

All data generated or analyzed during this study are included in this published article [and its supplementary information files].

## Abbreviations

TNBC: Triple-negative breast cancer
ER: Estrogen receptor
PR: Progesterone receptor
HER2: Human epidermal growth factor receptor 2
PDGF: Platelet-derived growth factor
hu-CB-BRGS: Humanized BALB/c-Rag2^null^Il2rγ^null^SIRPα^NOD^ mice
HIS: Human immune system mice
TILs: Tumor infiltrating lymphocytes
PD-1: Programmed death receptor-1
PD-L1: Programmed death-ligand 1
CTLA-4: Cytotoxic T-lymphocyte-associated protein-4
MHC: Major histocompatibility complex
DC: Dendritic cell
NK: Natural killer cell
TAM: Tumor associated macrophages
MDSC: Myeloid-derived suppressor cells
Treg: Regulatory T cell
GrB: Granzyme B
LN: Lymph node
EMT: Epithelial to mesenchymal transition
TGI: Tumor growth inhibition
ATAC-seq: Assay for transposase-accessible chromatin-sequencing
