# Supplementary figures and images for: RX-5902, a novel β-catenin modulator, potentiates the efficacy of immune checkpoint inhibitors in preclinical models of triple-negative breast Cancer
Source: BMC Cancer. 2020 Nov 4;20:1063. doi: 10.1186/s12885-020-07500-1 (PMC7641792; doi:10.1186/s12885-020-07500-1)

## Slide 1
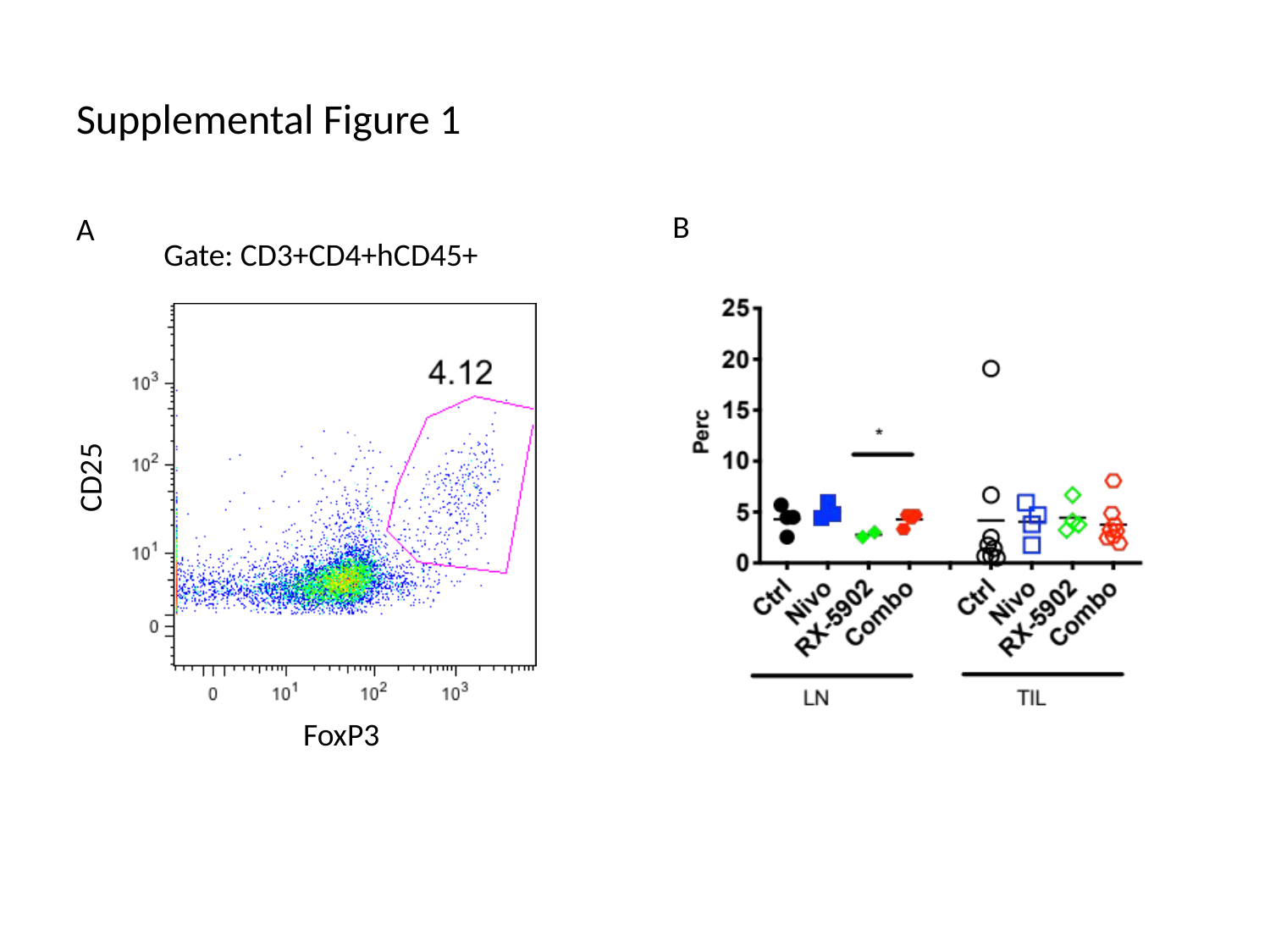

# Supplemental Figure 1
B
A
Gate: CD3+CD4+hCD45+
CD25
FoxP3

Supplement: Supplementary file 1 — Additional file 1: Figure S1. No difference observed in frequency of T regulatory cells (CD25 + FoxP3+) among human CD4+ T cells in the tumors of control or treated mice. A) Representative flow plots illustrating detection of Tregs by CD25 and FoxP3 and B) frequency of Tregs among CD4+ T cells in the lymph nodes (LN) and tumors (TIL) for each mouse, according to treatment. Bars represent arithmetic means. [file 12885_2020_7500_MOESM1_ESM.pptx]
