# Supplementary material for: RX-5902, a novel β-catenin modulator, potentiates the efficacy of immune checkpoint inhibitors in preclinical models of triple-negative breast Cancer
Source: BMC Cancer. 2020 Nov 4;20:1063. doi: 10.1186/s12885-020-07500-1 (PMC7641792; doi:10.1186/s12885-020-07500-1)
Supplement: Supplementary file 2 — Additional file 2: Table S1. Antibodies used in these studies. Antibody Sources, Clone ID and Fluorochromes are as indicated. [file 12885_2020_7500_MOESM2_ESM.docx]

| **Target**  Supplemental Table 1 | **Source** | **Clone** | **Fluorochrome** |  |  |
| --- | --- | --- | --- | --- | --- |
| CD4 | Biolegend | OKT4 | Fitc |  |  |
| CD5 | Biolegend | UCHT2 | Fitc |  |  |
| CD28 | Biolegend | CD28.2 | Fitc |  |  |
| CD33 | Biolegend | P67.6 | Fitc |  |  |
| Epcam | Biolegend | 9C4 | Fitc |  |  |
| Granzyme B | Biolegend | QA16A02 | Fitc |  |  |
| CD3 | Biolegend | HIT3a | PE |  |  |
| CD8 | Biolegend | Hit8a | PE |  |  |
| CD4 | Biolegend | OKT4 | PE |  |  |
| CD11c | Biolegend | s-hcl-3 | PE |  |  |
| CD69 | Biolegend | FN50 | PE |  |  |
| CD152 | Biolegend | BN13 | PE |  |  |
| CD3 | Biolegend | Hit3a | PERCP |  |  |
| CD4 | Biolegend | OKt4 | PERCP |  |  |
| CD20 | Biolegend | 2H7 | PERCP |  |  |
| hCD45 | Biolegend | HI30 | PERCP |  |  |
| CXCR3 | Biolegend | G025H7 | PERCP |  |  |
| HLA-A,B,C | Biolegend | w6/32 | PERCP |  |  |
| CD3 | Biolegend | Hit3a | PE Cy7 |  |  |
| CD11b | Biolegend | ICRF44 | PE Cy7 |  |  |
| hCD45 | Biolegend | HI30 | PE Cy7 |  |  |
| hIFNγ | Biolegend | 4S.B3 | PE Cy7 |  |  |
| hCD45 | Biolegend | HI30 | Pacific Blue |  |  |
| Tim3 | Biolegend | F38-2E2 | BV421 |  |  |
| CD25 | Biolegend | M-A251 | BV421 |  |  |
| CD274/PDL1 | Biolegend | 29E.2A3 | BV421 |  |  |
| CD19 | Biolegend | HIB19 | APC |  |  |
| CD279/PD1 | Biolegend | Eh12.2H7 | APC |  |  |
| HLA-DR | Biolegend | L243 | APC |  |  |
| TNFa | Biolegend | Mab11 | APC |  |  |
| FoxP3 | Invitrogen | 236A/E7 | APC |  |  |
| Tbet | Biolegend | 4B10 | APC |  |  |
| Granzyme B | Biolegend | QA16A02 | APC |  |  |
| mCD45 | Biolegend | 30-F11 | APCCy7 |  |  |
| hCD69 | Biolegend | FN50 | APCCy7 | ?? |  |
| HLA-DR | Biolegend | L243 | APCFire |  |  |
| CD8 | Biolegend | RPA-T8 | APCFire |  |  |
| FCR Block | Miltenyi |  |  |  |  |
| CD32 | BD Bioscience | 2.4G2 |  |  |  |
| mouse anti-human IgM UNLB | Southern Biotech | SA-DA4 |  |  |  |
| mouse anti-human IgG | Southern Biotech | JDC-10 |  |  |  |
| mouse anti-human IgM AP | Southern Biotech | UHB | AP |  |  |
| mouse anti-human IgG FC-AP | Southern Biotech | H2 | AP |  |  |
| Golgi Stop | BD Bioscience |  |  |  |  |
| cell stim | Invitrogen |  |  |  |  |
| saponin | Sigma |  |  |  |  |
| formaldehyde | Fisher |  |  |  |  |
| recombinant human IL6 | R&D systems |  |  |  |  |
| recombinant human SCF c-kit ligand | R&D systems |  |  |  |  |
| recombinant human FLT3 ligand | R&D systems |  |  |  |  |
| Bovine Serum Albumin | Sigma |  |  |  |  |
| FCS | Gibco |  |  |  |  |
| HBSS | Gibco |  |  |  |  |
| IMDM | Gibco |  |  |  |  |
| IgM from Human Sera | Sigma |  |  |  |  |
| IgG from Human Sera | Sigma |  |  |  |  |
| Zombie Green | Biolegend |  |  |  |  |
| Ghost Dye Red 780 | Tonbo |  |  |  |  |
|  |  |  |  |  |  |
|  |  |  |  |  |  |
|  |  |  |  |  |  |
|  |  |  |  |  |  |
|  |  |  |  |  |  |
|  |  |  |  |  |  |
|  |  |  |  |  |  |
|  |  |  |  |  |  |
|  |  |  |  |  |  |
|  |  |  |  |  |  |
|  |  |  |  |  |  |
|  |  |  |  |  |  |
|  |  |  |  |  |  |
|  |  |  |  |  |  |
|  |  |  |  |  |  |
|  |  |  |  |  |  |
|  |  |  |  |  |  |
|  |  |  |  |  |  |
|  |  |  |  |  |  |
|  |  |  |  |  |  |
|  |  |  |  |  |  |
|  |  |  |  |  |  |
|  |  |  |  |  |  |
|  |  |  |  |  |  |
|  |  |  |  |  |  |
|  |  |  |  |  |  |
|  |  |  |  |  |  |
|  |  |  |  |  |  |
|  |  |  |  |  |  |
|  |  |  |  |  |  |
|  |  |  |  |  |  |
|  |  |  |  |  |  |
|  |  |  |  |  |  |
|  |  |  |  |  |  |
|  |  |  |  |  |  |
|  |  |  |  |  |  |
|  |  |  |  |  |  |
|  |  |  |  |  |  |
|  |  |  |  |  |  |
|  |  |  |  |  |  |
|  |  |  |  |  |  |
|  |  |  |  |  |  |
|  |  |  |  |  |  |
|  |  |  |  |  |  |
|  |  |  |  |  |  |
